# Corticotropin-releasing factor induces functional and structural synaptic remodelling in acute stress

**DOI:** 10.1038/s41398-021-01497-2

**Published:** 2021-07-07

**Authors:** Dorien Vandael, Keimpe Wierda, Katlijn Vints, Pieter Baatsen, Lies De Groef, Lieve Moons, Vasily Rybakin, Natalia V. Gounko

**Affiliations:** 1 VIB-KU Leuven Center for Brain & Disease Research, Electron Microscopy Platform & VIB-Bioimaging Core, O&N5 Herestraat 49,, box 602, 3000 Leuven, Belgium; 2 KU Leuven Department of Neurosciences, Leuven Brain Institute, O&N5 Herestraat 49,, box 602, 3000 Leuven, Belgium; 3 VIB-KU Leuven Center for Brain & Disease Research, Electrophysiology Expertise Unit, O&N5 Herestraat 49, 3000 Leuven, Belgium; 4 KU Leuven Faculty of Science, Department of Biology, Laboratory of Neural Circuit Development and Regeneration, Naamsestraat 61, 3000 Leuven, Belgium; 5 National University of Singapore, Department of Microbiology and Immunology, Yong Loo Lin School of Medicine, and Immunology Program, 5 Science Drive 2, Blk MD4, 117545 Singapore, Singapore

**Keywords:** Hippocampus, Physiology

## Abstract

Biological responses to stress are complex and highly conserved. Corticotropin-releasing factor (CRF) plays a central role in regulating these lifesaving physiological responses to stress. We show that, in mice, CRF rapidly changes Schaffer Collateral (SC) input into hippocampal CA1 pyramidal cells (PC) by modulating both functional and structural aspects of these synapses. Host exposure to acute stress, in vivo CRF injection, and ex vivo CRF application all result in fast de novo formation and remodeling of existing dendritic spines. Functionally, CRF leads to a rapid increase in synaptic strength of SC input into CA1 neurons, e.g., increase in spontaneous neurotransmitter release, paired-pulse facilitation, and repetitive excitability and improves synaptic plasticity: long-term potentiation (LTP) and long-term depression (LTD). In line with the changes in synaptic function, CRF increases the number of presynaptic vesicles, induces redistribution of vesicles towards the active zone, increases active zone size, and improves the alignment of the pre- and postsynaptic compartments. Therefore, CRF rapidly enhances synaptic communication in the hippocampus, potentially playing a crucial role in the enhanced memory consolidation in acute stress.

## Introduction

Stress is a fundamental homeostatic reaction to any stimulus [1, 2], which can biologically manifest itself as predominantly positive ‘eustress’ or negative ‘distress’ [3]. Acute stress is an instantaneous and precise reaction to internal and environmental factors [4–6]. Although the mechanisms involved in regulating stress responses are well documented for the hypothalamic-pituitary-adrenal (HPA) axis pathway, the effect of stress on other regions of the brain is still not well understood [7, 8]. Among the many hormones, neuropeptides, and mediators involved in the stress response, CRF stands out due to its dual systemic (hormonal) and central (neuromodulatory) roles [8–10]. Centrally, CRF acts as a neuromodulator of synaptic transmission, which can be rapidly and locally released and acts within milliseconds [7] by binding to two different G protein-coupled receptors: CRF-receptor (CRF-R) 1 and 2 [4, 7, 10]. Activation of these receptors can result in a comprehensive array of cellular effects depending on the brain region and the specific CRF-family ligand binding [8, 11]. This can explain the diversity of responses reported in different brain regions to the same stressor. In the hippocampus, a region known for its involvement in learning and memory processes, CRF is expressed by GABAergic interneurons, which innervate PCs in CA1 and CA3 [12, 13] expressing CRF-Rs in distinct subcellular regions [4, 14, 15]. The effects of stress on—hippocampus dependent—memory storage and consolidation are complex [4, 16–18]. Mild or short stress enhances hippocampal functioning by promoting synaptic strengthening and by augmenting frequency of miniature excitatory postsynaptic currents (mEPSCs) and glutamate release probability [7, 19], while profound and chronic stress has detrimental effects, manifesting in the reduction in dendritic complexity and spine density in the hippocampus [12]. This spine loss is associated with attenuation of both LTP and LTD, and correlates with reported memory defects [7, 20, 21]. CRF contributes to the initiation of those stress-induced neuronal changes [7, 12, 22, 23] in a dose-, time-, and context-dependent manner [4, 16, 24, 25]. Especially the timing of CRF exposure is crucial for learning and memory processes and might result in opposite effects [4, 16, 24]. For example, short-term CRF application increases LTP [26] while prolonged exposure impairs hippocampal LTP [27].

Previous studies on structural changes reported a decrease in spine number and the reduction of dendritic complexity of PCs in CA1 and CA3 after long-term exposure to CRF [23, 28, 29]. The underlying molecular pathways of CRF-dependent plasticity have been mostly studied in the presence of high CRF concentrations and using in vitro assays. However, the acute effect of CRF in a physiologically relevant concentration (<250 nm) [8, 30, 31] on synaptic architecture and function in the hippocampus remains elusive.

Here, we show that acute stress, CRF stereotactic injections in vivo, and application of CRF ex vivo induces spine maturation and increases spine density in mice. At the synapse level, we demonstrate that acute CRF increases the presynaptic vesicular pool size, increases synapse number, induces a redistribution of synaptic vesicles towards the active zone and increases alignment of pre- and postsynaptic compartments. In line with these structural changes, we found that CRF facilitates synaptic transmission and increases synaptic reliability. In addition, CRF enhances long-term synaptic plasticity, which requires reciprocal activation of both CRF-Rs. Taken together, this study provides evidence that CRF is a crucial player in shaping the cellular response of hippocampal CA1 PCs during acute stress.

## Materials and methods

### Animals

All animal experiments were approved by the KU Leuven Ethical Animal Welfare Committee (protocol P019/2017) and were performed following the Animal Welfare Committee guidelines of the KU Leuven, Belgium. Mice were housed in a pathogen-free facility under standard housing conditions. In total, 113 male C57BL/6 Jax mice (P18–20), 29 male Thy1-YFP-H line, B6.Cg-Tg(Thy1-YFP)HJrs/J (P21–23, Jackson Laboratory cat.# 003782) and 4 male C57BL/6J-Tg(Thy1-GCaMP6)GP4.12Dkim/J (P18–20, Jackson Laboratory cat # 028278) were used.

### Acute stress induction and stereotactic injections in vivo

Thy1-YFP-H mice were used for acute stress and stereotactic injections with 100 nM CRF. For acute stress, we used two paradigms: foot shock (FS) and predator odor (PO) [32, 33]. For PO, mice were transferred from their home cage to a clean cage and subsequently exposed to either PO (domestic cat urine/fur mixed with cotton wool) or ambient air (cotton wool, control) [34]. FS was performed as described before [35]. Briefly, control animals stayed in the home cage without any handling. Acute stress FS protocol was a 0.1 mA electrical stimulation for 2 seconds. Twenty minutes after the stimulus, mice were deeply anesthetized with a mixture of ketamine/xylazine and cardiac puncture was carried out for trunk blood collection. Blood plasma was stored for corticosterone (CORT) ELISA analysis. Brains were collected after transcardiac perfusion with 4% paraformaldehyde (PFA; EMS) in 0.1 M phosphate buffer (PB; EMS). From each animal, one hemisphere was used for spine analysis of the PCs dendrites in the proximal region of CA1-Stratum Radiatum (SR), the other hemisphere was used for *cfos* and corticotropin-releasing hormone *(crh)* mRNA in situ hybridization (ISH) experiments. All acute stress experiments and blood collection were done during the same time of day (controlled for circadian rhythm).

For stereotactic injections of CRF in PCs CA1 hippocampus, mice were anesthetized by isoflurane and placed in a stereotactic frame with sustained anesthesia during and post injection. 300 nl of 100 nM CRF with a rate of 10 nl/sec was unilateral injected using a Nanoject II Auto-Nanoliter Injector (Drummond) using stereotactic coordinates: AP-2, ML-1.8, D-1.5 mm. The other (noninjected) hemisphere was used as a control [36]. Animals were perfused with 4% PFA in 0.1 M PB, 20 min after the injection. Until sample collection, animals were kept under anesthesia. Brains were post-fixed at 4 °C overnight. The following day, 100-μm-thick vibratome sections were made and used for further processing (see below).

### Determination of hormone concentrations and ISH after acute stress

Plasma was separated from whole blood and stored at −80 °C until further sample processing. CORT plasma levels were quantified using a CORT ELISA kit (DE4164, Demeditec Diagnostics). Blood plasma was 1:20 diluted with standard solutions. Absorbance was determined at 450 nm (reading) and 620–630 nm (background subtraction) with a microtiter plate reader.

Basescope hybridization was performed with the Basescope Detection Reagent Kit v2-RED (Advanced Cell Diagnostics). Briefly, 14-μm-thick cryosections of fixed frozen Thy1-YFP-H hemispheres of control and stressed mice were made. Superfrost slides (Thermofisher) with sections were baked at 60 °C for one hour before dehydrating steps of ethanol. After pretreatment solution steps, sections were incubated with custom-synthesized Basescope probes (*cfos*, BA-Mm-Fos-3zz-st targeting 676–801 of NM_010234.3 or *crh*, BA-Mm-Crh-3zz-st targeting 752–893 of NM_205769.3, see Supplementary Materials) each targeting all predicted transcript variants, followed by amplifying hybridization processes. Between amplification steps, slices were washed with wash buffer. Finally, slides were incubated with Fast Red for 10 minutes at room temperature in the dark and counterstained with 50% hematoxylin before drying at 60 °C. Brightfield images were taken with a Marzhauser Express 2 slide scanner (Nikon) using a ×20 objective. After imaging, the layer of PCs CA1 from each section was used for probe quantification. Probe-positive areas and physical CA1 PC areas were manually segmented using Microscope Image Browser (MIB, University of Helsinki) [37]. Data have been expressed as probe-positive areas relative to PCs-occupied areas.

### Dendritic spine filling ex vivo

For dye filling experiments in hippocampal acute slices, C57BL/6Jax mice were used, as described [38]. Briefly, animals were anaesthetized using isoflurane. After decapitation, the brain was quickly removed and transferred into ice-cold cutting solution: 83 mM NaCl, 2.5 mM KCl, 1 mM NaH_2_PO_4_, 22 mM glucose, 26.2 mM NaHCO_3_, 0.5 mM CaCl_2_, 3.3 mM MgSO_4_, 72 mM sucrose (Sigma), pH 7.4 with 5% CO_2_/95% O2. 300 μm coronal slices were cut with a Leica VT1200 vibratome. Slices could recover in a 34 °C cutting solution for 35 min and for 30 min at room temperature (RT) prior to transfer into artificial cerebrospinal fluid (aCSF): 119 mM NaCl, 2.5 mM KCl, 1 mM NaH_2_PO_4_, 26 mM NaHCO_3_, 4 mM MgCl_2_, 4 mM CaCl_2_, 11 mM glucose at pH 7.4 with 5% CO_2_/95% O_2_. Glass borosilicate recording pipettes (resistance 3.5–5.5 MΩ) were filled with 10 mM Alexa 568 (Life Technologies) dissolved in internal solution: 115 mM CsMSF, 20 mM CsCl, 10 mM HEPES, 2.5 mM MgCl_2_, 4 mM ATP, 0.4 mM GTP, 10 mM creatine phosphate and 0.6 mM EGTA (Sigma Aldrich). Whole-cell configuration was used to fill CA1 PCs for 10–15 min in control slices and slices incubated with 100 nM CRF added to the aCSF for 20 min. Hence, slices are incubated 10 min prior to the filling with aCSF and CRF. Treatment with blockers was carried out by directly adding them to aCSF for 20 min before reaching whole-cell mode. For condition of blockers with CRF, CRF was added 10 min after slices were exposed to the specific CRF-R blockers. Sections were fixed with 4% PFA and 2% sucrose in 0.1 M PB at 4 °C overnight.

### Spine imaging and analysis ex vivo and in vivo

After 4% PFA fixation overnight, brain slices were washed three times with 0.1 M PB and mounted using mounting medium (Vectashield). Hundred-micrometers-thick vibratome sections were made from brains collected after acute stress paradigms and stereotactic injections of CRF, as described above. Secondary and tertiary dendrites of PCs in the proximal region of the CA1 were imaged with a Structured Illumination Microscopy (Elyra S.1, Zeiss) with a ×63 plan-apochromat 1.4 oil DIC objective. Images were processed using the Zeiss software. Dendritic protrusions were counted in Z-stack (Z-step of 0.025 µm) and quantified using ImageJ (NIH). We classified five spine types. Mushroom spines: possess a spine head of more than 0.5 µm. Stubby: length shorter than 1.0 µm. Spine head diameter larger than spine length. Thin: length shorter than 1.0 µm possessing, spine head diameter shorter than spine length. Long thin: length between 1.0 and 1.5 µm. Filopodia: longer than 1.5 µm.

### Electrophysiological and multi electrode array (MEA) ex vivo studies

#### Ex vivo

Acute slices (300 μm) were prepared from C57BL/6Jax mice the same way as for ex vivo spine fillings, as described before [38]. After recovery, brain slices were continuously perfused in a submerged chamber (Warner Instruments) at a rate of 3–4 ml/minutes with aCSF at pH 7.4 with 5% CO_2_/95% O_2_. Control slices and slices incubated with 100 nM CRF added to the aCSF for ~20 min before recording were used. For mEPSCs, coronal sections were prepared and 1 µM tetrodotoxin (TTX) was added to the aCSF. For paired-pulse recordings, train stimulation, and AMPA/NMDA characterization, sagittal slices were used and 20 μM bicuculline was added to the aCSF. Whole-cell patch-clamp recordings were done using borosilicate glass recording pipettes (resistance 3.5–5.5 MΩ) filled with a CsMSF-based internal solution (see ex vivo spine filling). Spontaneous input to CA1 PCs was recorded by whole-cell voltage-clamp recordings (Vm = −70 mV and Rs compensation was set at ~70%) from visually identifiable CA1 PCs, using a Multiclamp 700B amplifier (Axon Instruments) and analyzed using Mini Analysis program (Synaptosoft). For evoked recordings (Vm = −70 mV, Rs compensation ~70%), SCs were stimulated using A-M systems 2100 isolation pulse stimulator and a 2-contact cluster microelectrode (CE2C55, FHC) placed in SR at the border of CA1-CA2. For paired-pulse ratio analysis, paired extracellular stimulations (interstimulus interval (ISI): 25, 50, 100, 200, 400, and 1000 ms) were delivered every 20 seconds (each ISI was repeated three times) and peak amplitudes were calculated as the EPSC2/EPSC1 ratio. For train stimulations, 200 stimuli were delivered at the following frequencies: 2, 5, 10, and 20 Hz. Peak amplitudes and total charge were quantified and normalized to the first evoked response of the train. Peak AMPAR-mediated evoked EPSCs were measured in whole-cell voltage-clamp at a holding potential of −60 mV, while the NMDAR-mediated component was measured 100 ms after initiation of the combined AMPAR-and NMDAR-mediated EPSCs recorded at +40 mV. Measurements were performed in a minimum of three independent preparations.

#### MEA

Parasagittal slices (300 μm) were prepared from C57BL/6Jax mice and used for fEPSPs recording using commercially available MEAs, 87 electrodes in an 8 × 8 lay-out (MEA2100, Multi Channel Systems) as described before [39, 40]. The recording chamber was perfused with aCSF and maintained at 32 °C. A slice grid was put on the top of the slices to assure immobilization and optimal contact with electrodes. Data streams were sampled at 10 kHz. For each slice, a single electrode located underneath the SC pathway was visually selected for stimulation. Biphasic, constant voltage pulses (100 µs pulse width) were applied to evoke fEPSPs from the SC in the CA1. After establishing stable fEPSP signals (~30 min), an input/output curve was generated using stimulation intensities from 0.5 to 2.750 V (in steps of 0.25 V), each applied twice with 30–120 seconds interval was established. The stimulus intensity eliciting 35% of the maximal fEPSP amplitude was used for further stimulation.

Next, we recorded baseline fEPSPs for ~25 min (3 stimulations 15 seconds apart, every 3 min). For CRF conditions, after 5 min of baseline, we switched to aCSF with 100 nM CRF, recorded 15 min of baseline, switched back to aCSF which normalized a stable baseline comparable to before CRF application. After reestablishing a stable baseline, we either applied train stimulations (LTP) or low frequency stimulations (LTD). LTP was introduced by three trains of high-frequency stimulation at 100 Hz (100 stimuli at 100 Hz), with 5 min interval. For induction of LTD, low frequency stimulation of 1 Hz, 900 pulses was induced to introduce LTD in the CA1 region. Post-LTD or -LTP induction, fEPSPs were recorded for 65 minutes (3 stimulations 15 seconds apart, every 3 min).

### Calcium imaging in vivo

Acute coronal slices (300 μm) were prepared from Thy1-GCaMP6 mice (see above). After recovery, brain slices were continuously perfused with aCSF during the imaging of the CA1 at RT with a two-photon system (VIVO 2-Photon platform, Intelligent Imaging Innovations GmbH) using a ×20 objective. Imaging started in aCSF capturing 300 images of the region of interest (ROI), average of 15 frames per image, 30 ms intervals. 600 images were taken: 300 control aCSF images and another 300 images where CRF was present in the aCSF. After 15 min with CRF in aCSF, another 600 images were taken with the same settings in the same ROI.

### Electron microscopy (EM) and analysis

#### Ex vivo

Acute coronal slices (300 µm) were prepared from C57BL/6Jax mice (see above). After recovery, control and CRF-treated slices (100 nM CRF for 20 min) were fixed for at least 2 h at room temperature. For synaptic morphology we used 4% PFA, 2% glutaraldehyde (EMS, USA), 0.2% picric acid (EMS, USA) in 0.1 M PB, pH 7.4 For active zone (AZ) and postsynaptic densities (PSD) quantification, we used 4% PFA in 0.1 M PB, pH 7.4.

#### In vivo

Thy1-YFP-H mice were used for the acute stress foot shock (FS) paradigm. FS was performed as above. Twenty minutes after the stimulus, mice were deeply anesthetized with a mixture of ketamine/xylazine and cardiac puncture was carried out for trunk blood collection. Blood plasma was separated and reserved for corticosterone (CORT) ELISA analysis. Brains were collected after trancardiac perfusion with 4% PFA (EMS) in 0.1 M PB (EMS) and post-fixed at 4 °C overnight. The following day, 100-μm-thick vibratome sections were made and used for further processing (see below).

For synaptic morphology analysis with transmission electron microscopy (TEM), after fixation slices were subsequently washed with 0.1 M PB and 0.1 M cacodylate buffer and post-fixed for 60 min on ice in 0.1 M cacodylate buffer (EMS, USA) containing 1% OsO_4_ (EMS, USA) and 1.5% C_6_FeK_4_N_6_ (EMS, USA), pH 7.6. Next, slices were washed once with 0.1 M cacodylate buffer, and then with dH_2_O. The slices were contrasted with 0.5% uranyl acetate (EMS, USA) in 25% methanol at 4 °C overnight. The following day, slices were washed with dH_2_O and stained on bloc with Walton’s lead aspartate [38] at 60 °C for 30 min, and washed with dH_2_O. Afterwards, the samples were dehydrated in a graded series of ethanol solutions and were treated twice for 10 min with propylene oxide and infiltrated with medium Epon 812/propylene oxide mixtures. The next day, sections were flat embedded in medium composition of Epon 812 (EMS, USA) between two microscopic slides and ACLAR film (EMS) and polymerized for 2 days at 60 °C.

For visualization and analysis of AZ and PSD with TEM and focused ion beam scanning electron microscope (FIB-SEM), After fixation slices were washed with 0.1 M PB and dehydrated in a graded series of ethanol solutions. Afterward, slices were treated for 30 min at 60 °C in 1% ethanolic phosphotungstic acid (PTA; MP Biomedicals). Slices were washed with pure ethanol and subsequently with pure acetone. The slices were contrasted with 2% uranyl acetate in acetone at 60 °C for 20 min. Slices were then washed with acetone and incubated in 0.5% lead acetate in acetone at 60 °C for 20 min, washed with acetone and infiltrated with hard Epon 812/acetone mixtures. The next day, slices were embedded in hard composition of Epon 812 and polymerized for 2 days at 60 °C.

For TEM imaging ultrathin sections (70 nm) were collected on single slot copper grids and counterstained with uranyl acetate and lead citrate. Images of these sections were made at ×25k magnification for synaptic boutons morphology and at ×15k magnification for AZ/PSD analysis, using a TEM (JEM1400, Jeol) equipped with a SIS Quemesa (Olympus) camera operated at 80 kV.

For the FIB-SEM, the embedded samples were coated with ~8 nm platinum. FIB-SEM imaging is performed using a Crossbeam 540 (Zeiss) system with Atlas 3D software. The FIB-SEM was used to remove a 5-nm-thick layer by propelling gallium ions at the surface of the specimen. Image acquisition was done at 1.5 kV (0.005 µm/pixel) using a backscattered electron detector, at ×5k magnification. Images were aligned with Atlas 3D software.

PCs CA1 synapses were identified by their morphology and localization. Image segmentation of individual pre- and postsynaptic terminals, PSDs, AZs and synaptic vesicles was performed initially by using MIB software. For vesicle analysis, we estimated the shortest straight path connecting the center of vesicle to the AZ and calculated the smallest angles between the directions of this path. The statistics for synaptic surface area, AZ/PSD area and length, number of vesicles and distance from AZ was collected using a custom-made script in ImageJ. Amira software was used for visualization of AZs and PSDs in 3D.

### Chemicals and treatments

Alexa 568 hydrazide was used at—10 mM (Thermo Fisher Scientific), Antisauvagine-30 (aSvg)—150 nM (Tocris), bicuculline—20 μM (Sigma Aldrich), CRF—100 nM (Bachem), NBI 27914 (NBI)—1.2 µM (Tocris), TTX—1 µM (Tocris). Besides Alexa 568, all compounds were dissolved in DMSO prior to dilution into appropriate aqueous buffers/solutions.

### Quantification and statistical analysis

Data analysis was carried out in ImageJ (NIH), Clampfit (Molecular devices), Mini Analysis (Synaptosoft), Multi channel analyzer software (Multi channel systems), Microscope Image Browser (MIB, University of Helsinki), Amira (Thermo Scientific), Atlas 3D (Zeiss), and Excel (Microsoft). Data statistic was carried out in GraphPad Prism 8 (GraphPad software).

We did not calculate sample size for ensuring adequate power or randomization of the samples. Some analysis was performed blindly (e.g., spine density, ISH data). Animals and brain sections with a deteriorated general health were excluded from the study.

We first evaluated the quantitative sample distributions for normality using the D’Agostino-Pearson test. Subsequently, either Mann–Whitney test (for non-normal distributions) or unpaired *t*-test (for normal distributions) was used to compare statistical differences between two groups. Multiple group comparisons were performed with the Kruskal–Wallis analysis of variance (ANOVA) followed by Dunn’s multiple comparison test (for non-normal distributions) or with one-way ANOVA followed by Dunnett’s multiple comparison test (for normal distributions). Normal distributions are represented as the mean with the standard error of the mean (±SEM) while non-normal distributions are represented as the median with interquartile range (IQR). Results were evaluated at a 5% significance level. Sample size and statistical tests (including the *p*-values) used for each comparison are detailed in the figure legends.

## Results

### Both short-term stress and CRF treatment induce spine formation in vivo

**Fig. 1:**
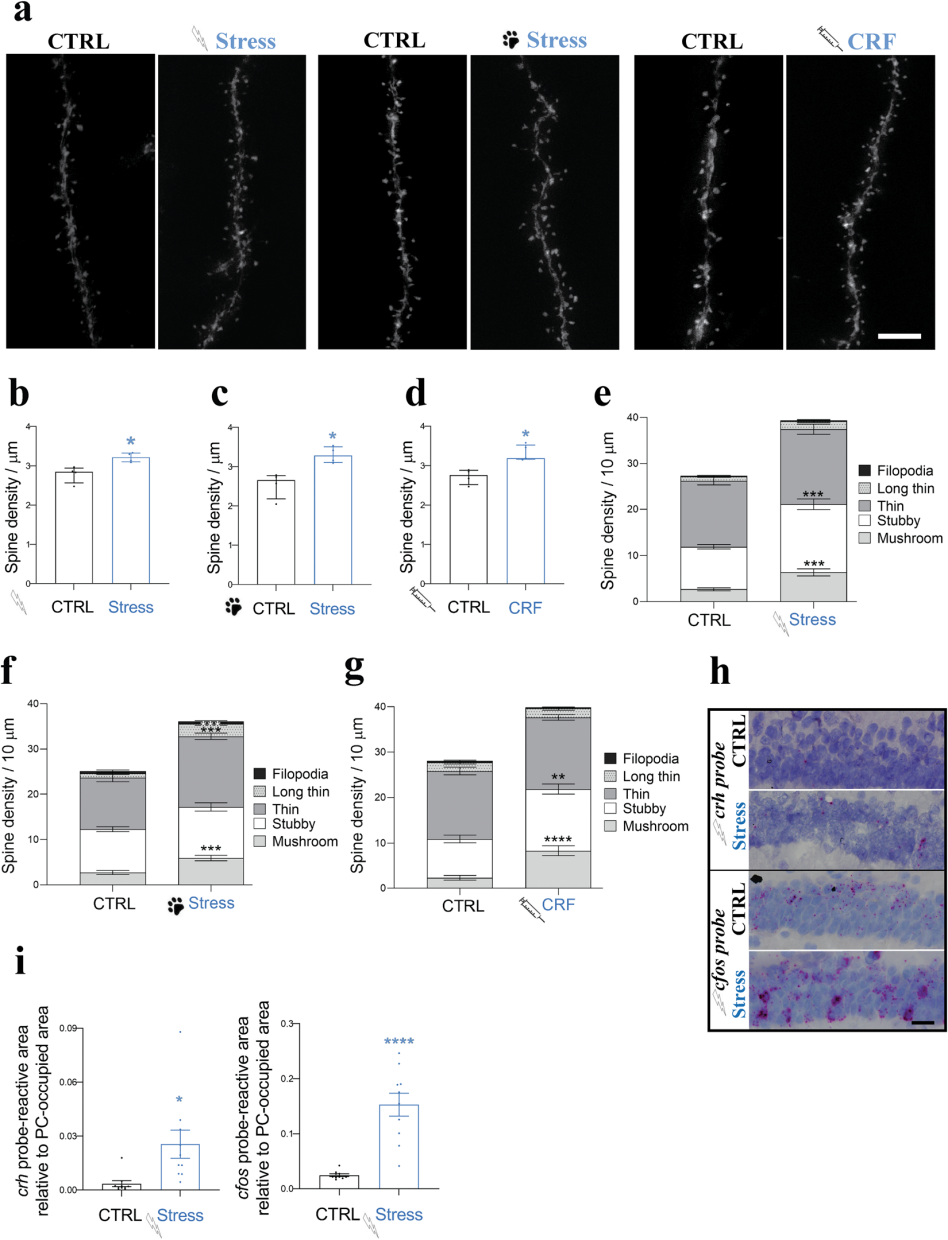
Acute stress and CRF increase spine density in CA1 hippocampus in vivo. **a** Images of CA1 PC dendrites from Thy1-YFP mice before and after acute stress. Left two images: foot shock (FS) paradigm, middle two images: predator odor (PO) paradigm, right two images: 20 min 100 nM CRF treatment using stereotactic injections into the hippocampal formation. Scale bar = 5 µm. **b**–**d** Quantification of spine densities after FS, PO, and CRF treatment (shown as median with IQR, CTRL: *N* = 4 animals, *n* = 34 dendrites; FS: *N* = 4, *n* = 34; PO: *N* = 4, *n* = 31; acute CRF treatment: *N* = 5, *n* = 34; Mann–Whitney tested (U = 0); *p < 0.05). **b** Acute stress paradigms FS, **c** PO and **d** acute CRF treatment increase spine density. **e**–**g** Quantification of spine types. **e** Acute stress paradigms FS (shown as mean ± SEM, CTRL: *N* = 4, *n* = 19; FS: *N* = 5, *n* = 18; multiple *t*-test (filopodia; t ratio = 0.6804, long thin; t ratio = 2.696, thin; t ratio = 1.513, stubby; t ratio = 4.728, mushroom; t ratio = 4.363). ***p < 0.0001). **f** PO (shown as mean ± SEM, CTRL: *N* = 5, *n* = 18; PO: *N* = 4, *n* = 18; multiple *t*-test filopodia; t ratio = 0.2124, long thin; t ratio = 4.100, thin; t ratio = 4.141, stubby; t ratio = 0.9460, mushroom; t ratio = 4.868). ***p < 0.0005) and **g** acute CRF treatment (shown as mean ± SEM, CTRL: *N* = 3, *n* = 14; CRF injections: *N* = 3, *n* = 15; multiple *t*-test filopodia; t ratio = 0.8867, long thin; t ratio = 0.2817, thin; t ratio = 0.9384, stubby; t ratio = 3.645, mushroom; t ratio = 4.784). **p < 0.005) promote spine maturation in PCs CA1. **h** FS increases *crh* (left) and *cfos* (right) mRNA expression in CA1 PCs. Scale bar = 25 µm. **i** Quantification of *crh* (left) (shown as mean ± SEM from FS CTRL: *N* = 4, *n* = 10 sections; FS: *N* = 4, *n* = 10; unpaired *t*-test (t = 2.765, df = 18). *p < 0.05) and *cfos* (right) mRNA expression (shown as mean ± SEM from FS CTRL: *N* = 3, *n* = 10 sections; FS: *N* = 3, *n* = 10; unpaired *t*-test (t = 6.122, df = 18). ****p < 0.0001).

Previous studies in different brain regions have shown that stress induces changes in spine density and morphology [41–44]. To investigate the effect of short-term stress on spines of hippocampal CA1 PCs in vivo, we compared two independent models for acute, mild stress in mice expressing YFP in CA1 PCs: foot shock (FS) and predator odor (PO). CORT levels were mildly elevated in blood plasma 20 min after FS and PO acute stress paradigms (Supplementary Fig. 1a, b). These data fit with the initial phase of the stress response, since plasma CORT levels have been reported to significantly increase only 30 min to an hour after stress induction [45–47]. In both paradigms, we found a significant increase in spine density compared to unstressed animals (Fig. 1a–c). In addition, acute stress using the FS paradigm shifts spine morphology towards more mature types (Fig. 1e, d): mushroom and stubby [48, 49]. In PO experiments, both the increase in spine density and the shift in spine morphology (Fig. 1f) were less prominent compared to FS. Since acute stress-induced changes in CORT levels in the hippocampus and other brain regions takes at least 30 min [50, 51], a systemic component is very unlikely to be involved in the structural changes in spine density and morphology we find within 20 min after acute stress induction.

Next, we performed stereotactic injections of CRF into CA1 of YFP-expressing mice to determine whether CRF has the same effect on spines as acute stress. Indeed, CRF significantly increased spine density compared with control (Fig. 1a, d) and induced a shift in spine morphology towards more mature types (mushroom and stubby), comparable to the acute stress paradigms (Fig. 1e–g).

To confirm direct stress response in the hippocampus, we performed ISH analysis for immediate early genes *crh* and *cfos* [52–54], in mice 20 min after being subjected to FS. We observed a local increase of *crh* and *cfos* mRNA expression in the CA1 PC layer (Fig. 1h, i), demonstrating an upregulation of immediate early genes in the CA1 PC layer directly after acute stress.

### Acute CRF exposure increases the spine density of CA1 PCs

**Fig. 2:**
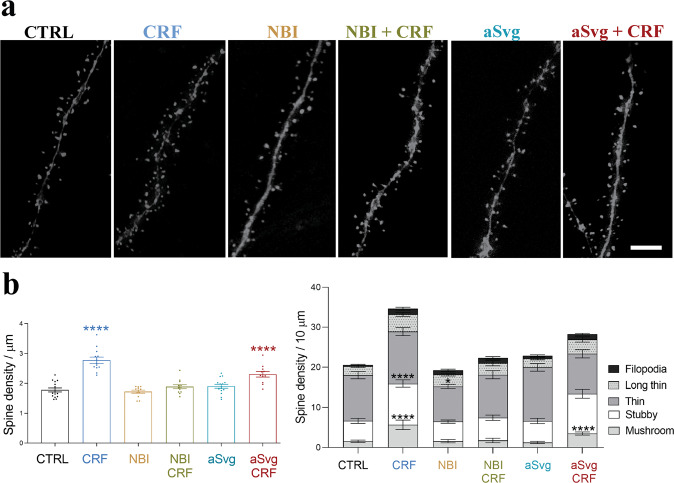
Short-term CRF application increases pyramidal cell spine density and maturation ex vivo. **a** Spines on CA1 PC dendrites filled with Alexa 568 using: no treatment (CTRL), only 100 nM CRF for 20 min, selective CRF-R1 antagonist NBI (1.2 μM, NBI), combined NBI and CRF (NBI + CRF), selective CRF-R2 antagonist aSvg (150 nM, aSvg) and combined aSvg and CRF (aSvg + CRF) application. Scale bar = 5 µm. **b, c** Quantification of spine densities (**b**, shown as mean ± SEM, CTRL; *N* = 3 animals, *n* = 15 cells; CRF: *N* = 5, *n* = 13; NBI: *N* = 4, *n* = 12; NBI + CRF: *N* = 5, *n* = 12; aSVG: *N* = 5, *n* = 15; aSVG + CRF: *N* = 4, *n* = 9; one-way ANOVA with Dunnett’s multiple comparisons test (F = 21.25). *p < 0.05, ****p < 0.0001), and type (**c**, shown as mean ± SEM; two-way ANOVA with multiple comparisons (F = 21.25). *p < 0.05, ***p < 0.005, ****p < 0.0001) using aforementioned conditions.

To allow a more detailed analysis of the molecular pathway and functional consequences of acute CRF exposure in CA1 PCs, we investigated if the effect of direct CRF injections on spines can be recapitulated in acute hippocampal slices. Indeed, short-term CRF application significantly increased spine density and maturation of dye-filled PCs in acute hippocampal slices (Fig. 2a, b).

Using acute slices, we set out to identify the underlying CRF receptors involved in mediating the acute spine changes, by pretreating acute slices with their selective antagonists (CRF-R1:NBI 27914 (NBI); K_i_ = 1.7 nM, CRF-R2: Antisauvagine-30 (aSvg); K_i_ = 1.4 nM) immediately before CRF treatment. Application of either antagonist alone did not significantly affect spines of CA1 PCs (Fig. 2a, b). Inhibition of CRF-R1s completely blocked the CRF-induced increase in spine density and maturation (Fig. 2a–c), while inhibition of CRF-R2 partially blocked this CRF effect. Together, these data show that the acute CRF-induced increase in mature spine number is predominantly dependent on CRF-R1 signaling, although CRF-R2s can play a complementary role, potentially requiring the deployment of calcium stores [55].

The changes in spine density and maturation sustained at least 2 h after the removal/wash out of CRF, suggesting these are long-lasting structural modifications (Supplementary Fig. 2).

### Acute CRF exposure modulates functional properties of SC input into CA1

**Fig. 3:**
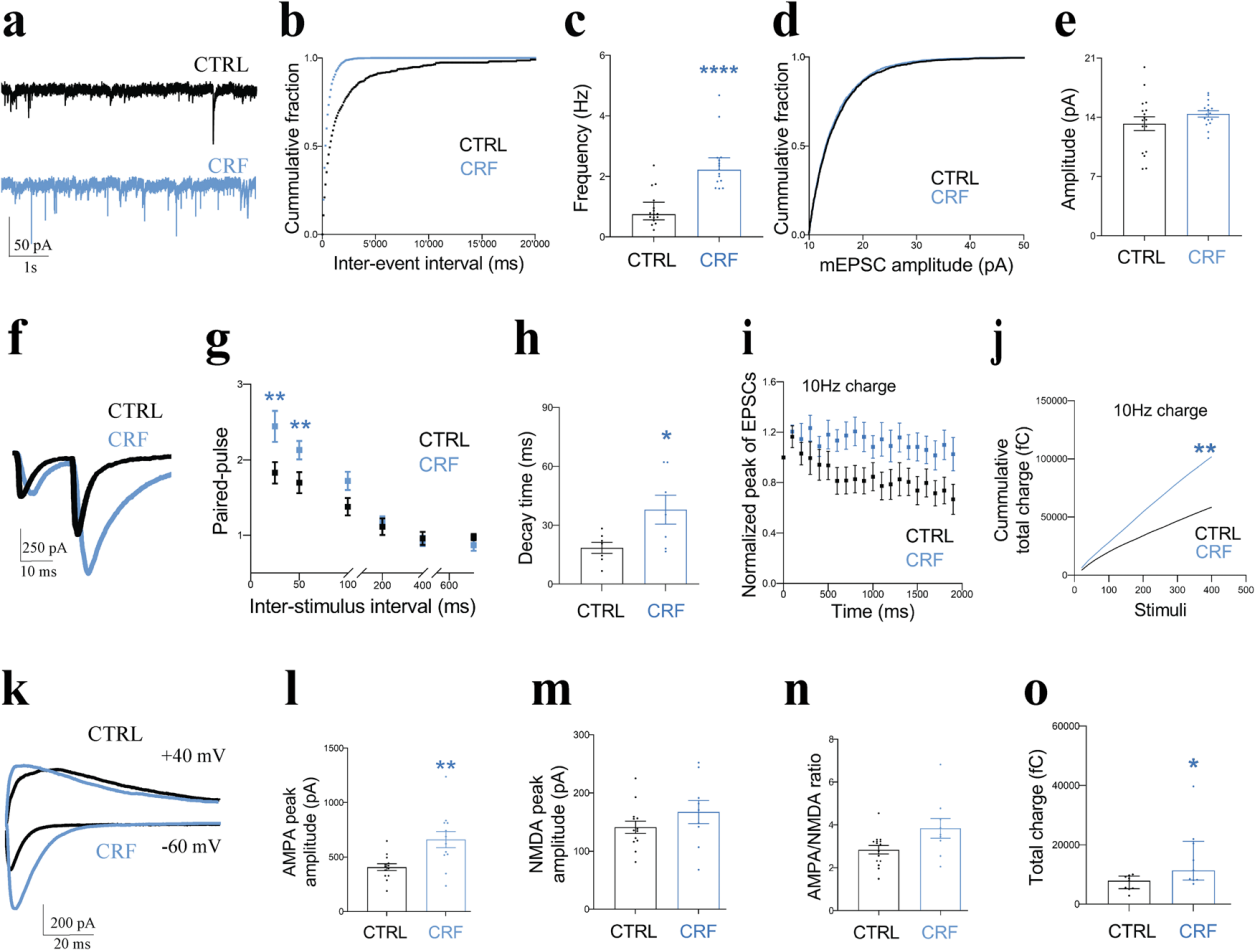
Acute CRF treatment increases synaptic input and synaptic reliability. **a** mEPSCs recorded in CA1 PCs in control (black) and 20 min after 100 nM CRF treatment (blue). **b, c** CRF increased mEPSCs frequency (shown as median with IQR. CTRL: *N* = 3 animals, *n* = 16 cells; CRF: *N* = 3, *n* = 15; Mann–Whitney test (U = 15). ****p < 0.0001), **d, e** but not amplitude (shown as mean ± SEM. CTRL: *N* = 3, *n* = 17; CRF: *N* = 4, *n* = 16; unpaired *t*-test (t = 1.267, df = 31). *P* = 0.2147). **f** Stimulation of SCs resulting paired-pulse input in recorded CA1 PCs under control (black) and CRF pretreated conditions (blue). **g, h** CRF increased amplitude, increased paired-pulse facilitation with 25 and 50 ms inter-stimulation intervals (**g**, shown as mean ± SEM. CTRL: *N* = 4, *n* = 15; CRF: *N* = 4, *n* = 16; unpaired *t*-test (for 25 ms; t = 3.406 and df = 31, for 50 ms; t = 2.835 and df = 31). **p < 0.01), and the decay time (**h**, shown as mean ± SEM. CTRL: *N* = 4, *n* = 15; CRF *N* = 4, *n* = 16; unpaired *t*-test (t = 3.738 and df = 31). ***p < 0.001) of the second evoked EPSC. **i, j** Normalized EPSC amplitude **i** and **j** cumulative total charge released during train stimulation (10 Hz, 200 stimuli) in control (black) and CRF-treated (blue) (shown as median with IQR. CTRL: *N* = 5, *n* = 17; CRF: *N* = 6, *n* = 15; Mann–Whitney test (U = 58). **p < 0.01). **k, l** CRF increased AMPAR-mediated EPSC amplitude at SC-CA synapses (Vm = −60 mV, black) (**l**, shown as mean ± SEM. CTRL: *N* = 4, *n* = 13; CRF: *N* = 3, *n* = 12; unpaired *t*-test (t = 3.189, df = 23). **p < 0.005), but not NMDAR-mediated EPSC amplitude (Vm = +40 mV, blue), (**m**, shown as mean ± SEM. CTRL: *N* = 4, *n* = 13; CRF: *N* = 3, *n* = 9; are unpaired *t*-test (t = 1.257, df = 20). *P* = 0.2234). **n** AMPAR/NMDAR ratio (shown as mean ± SEM. CTRL: *N* = 4, *n* = 15; CRF: *N* = 3, *n* = 9; unpaired *t*-test (t = 2.319, df = 22). *p < 0.05). **o** CRF increases total charge transfer during AMPAR-mediated EPSCs (shown as median with IQR. CTRL: *N* = 4, *n* = 9; CRF: *N* = 3, *n* = 9; Mann–Whitney test (U = 17). *p < 0.05).

To determine if the CRF-induced increase in (mature) spine density translates into enhanced functional synaptic connections, we set out to study synaptic function, starting with recording mEPSCs in CA1 PCs. Using acute slices exposed to 100 nM CRF for 15 min prior to the start of and throughout the recordings, we showed a robust increase in mEPSC frequency, but not amplitude (Fig. 3a–e). This finding suggests an increase in the number of excitatory synapses, or an increase in release probability of individual synaptic connections. We already found a CRF-induced increase in mature spine density, in line with an increase in functional synaptic connections. However, we also observed ultrastructural changes within synapses, which are in line with an increase in release probability (see below). In addition, CRF-induced enhanced network activity as evident from our calcium imaging of CA1 PCs in ex vivo acute slices from mice expressing the fluorescent calcium indicator, GCaMP6s (Supplementary Fig. 3, Supplementary Video 1). Together, these data suggest that the CRF-induced increase in mEPSC frequency is due to a combination of structural and functional adaptations.

To explore alterations of presynaptic release probability in more detail, we used electrical stimulations of the SC pathway projecting onto the CA1 PCs. Using paired-pulse stimulations, we found increased paired-pulse facilitation (PPF) with 25 ms and 50 ms intervals in the presence of CRF (Fig. 3f–j), suggesting a change in the functional organization of SC presynaptic terminals. We observed a striking increase in the decay time constant in CRF-treated slices (Fig. 3h), suggesting increased sustained/asynchronous release following evoked release. To further explore the effect of acute CRF exposure during more demanding periods of SC input activity, we performed train stimulations and analyzed both the synchronous peak amplitude and the total cumulative evoked charge. We observed decreased synaptic fatigue during 10 Hz train stimulations (Fig. 3i) and an almost two-fold increase in the absolute total cumulative charge after CRF treatment (Fig. 3j). Together, these observations suggest that CRF changes presynaptic function, ultimately resulting in enhanced synaptic reliability.

To determine if CRF indeed affects the number of mature/functional synaptic contacts (as suggested by mEPSC frequency and changes in spines), we stimulated SC inputs and consecutively recorded AMPA- and NMDA-receptor mediated evoked EPSCs (−60 and +40 mV, respectively, Fig. 3k–n). CRF treatment induced a significant increase in AMPA component, both amplitude and total charge (Fig. 3l, o), while NMDA amplitude was unaltered. Consequently, CRF increased the AMPA/NMDA ratio, suggesting a shift towards mature/functional synaptic connections, in line with our spine analysis data.

**Fig. 4:**
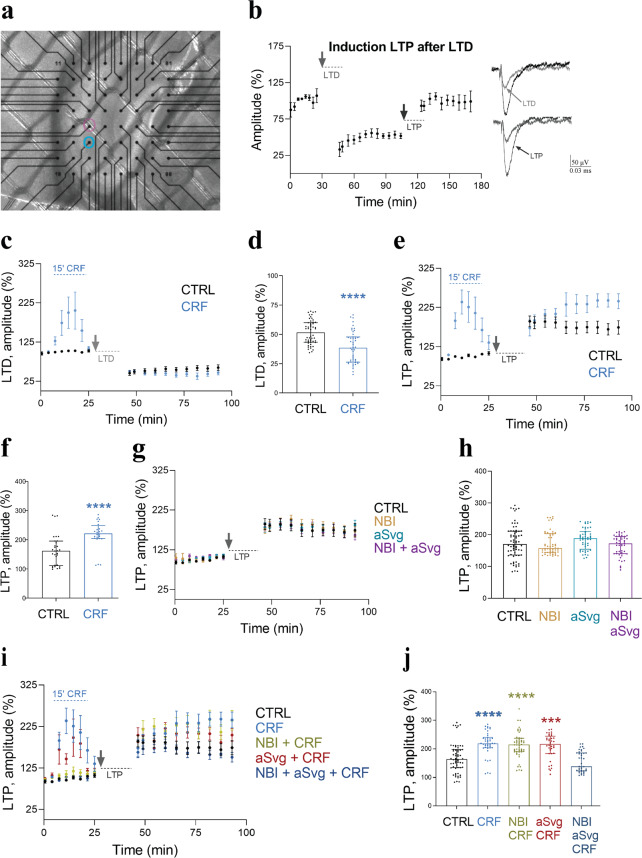
CRF can augment long-term synaptic plasticity via CRF-R1 or CRF-R2 activation. Multi electrode array recording of evoked fEPSP from the SC in the CA1. **a** Image of a mouse acute hippocampal slice on the multi electrode array (MEA2100; Multi channel Systems) used for field excitatory postsynaptic potential (fEPSP) recordings with the stimulation electrode (blue) and recording electrode (pink) to stimulate Shaffer collateral-CA1 connections. **b** Consecutive long-term depression (LTD) and long-term potentiation (LTP) induction. Baseline fEPSPs were recorded for approximately 25 min, LTP induction protocol was applied to the same slices and recording continued for another 60 min. **c** LTD in control slices (black) and slices treated with CRF (15 min, 100 nM CRF). Treatment period indicated with dashed line (blue). **d** Averaged fEPSC amplitude 60 min after LTD induction (normalized to baseline), (shown as median with IQR. CTRL: *N* = 9 animals; CRF: *N* = 8; Mann–Whitney test (U = 631). ****p < 0.0001). CRF treatment increased LTD by 17% compared to control. **e** LTP in control slices (black) and slices treated with 100 nM CRF (blue) for 15 min. Treatment period indicated with dashed line. **f** CRF increased LTP efficiency by 32% (shown as median with IQR. CTRL: *N* = 11 animals; CRF: *N* = 9; Mann–Whitney test (U = 139). ****p < 0.0001). **g** LTP induction in combination with either CRF-R1 blocker (NBI 1.2 µM), CRF-R2 blocker (aSvg 150 nM) or both. **h** CRF-R blockers do not affect LTP (shown as median with IQR. CTRL: *N* = 11; NBI: *N* = 9; aSVG: *N* = 8; NBI + aSVG: *N* = 8; Kruskal–Wallis test with Dunn’s multiple comparisons test (Kruskal–Wallis statistic = 6.357). *P* > 0.9999, *p* = 0.6285, *p* > 0.9999). **i** LTP induction in the presence of CRF-Rs blockers. Blockers were present throughout the recording. **j** Effect of CRF on LTP can be established via both CRF-Rs pathway, but no additivity was found if both pathways are available (shown as median with IQR. CTRL: *N* = 11; CRF: *N* = 9; NBI: *N* = 9; NBI + CRF: *N* = 9; aSVG: *N* = 8; aSVG + CRF: *N* = 8; NBI + aSVG: *N* = 8; NBI + aSVG + CRF: *N* = 8; Kruskal–Wallis test with Dunn’s multiple comparisons test (Kruskal–Wallis statistic = 94.32). ***p < 0.0005, ****p < 0.0001).

To explore the long-term effects of CRF on synaptic plasticity and network function, we examined LTD and LTP of the SC pathway onto CA1 PCs, using MEA extracellular field potential recordings (field excitatory postsynaptic potentials, fEPSPs) (Fig. 4). In the cerebellum, LTD induction requires CRF [56], but this CRF-dependency of LTD generation has not been reported in the hippocampus [57–59]. First, we confirmed that we were able to induce substantial LTD and subsequently LTP on the same acute slices (Fig. 4b). Next, we investigated the effect of acute CRF application on baseline fEPSP amplitude and subsequently on LTD or LTP in separate experiments (Fig. 4c–f). During CRF application (15 min, indicated with “15’ CRF”, Fig. 4c, e, g) we observed a clear increase in fEPSP amplitude, likely representing the short-term increase in synaptic function/reliability described above. This increase in fEPSP was transient and after CRF wash out, the amplitude returned to baseline, as previously described [26]. Intriguingly, CRF treatment significantly enhanced LTD and LTP induction (Fig. 4d, f), seemingly increasing the spectrum and/or sensitivity of long-term plasticity mechanisms. To determine the involvement of CRF-Rs in enhancing LTP, we combined application of the CRF-receptor antagonists NBI and aSvg with LTP induction. By themselves, these blockers did not affect baseline fEPSP amplitudes or LTP induction (Fig. 4g). Combined with CRF treatment, blocking either of the two CRF-Rs did not inhibit CRF-induced enhancement of LTP (Fig. 4h, i). However, combining both blockers abolished the acute CRF-dependent LTP enhancement, indicating that activation of either receptor is sufficient for this form of plasticity.

### Acute CRF exposure leads to ultrastructural alterations of synapses

**Fig. 5:**
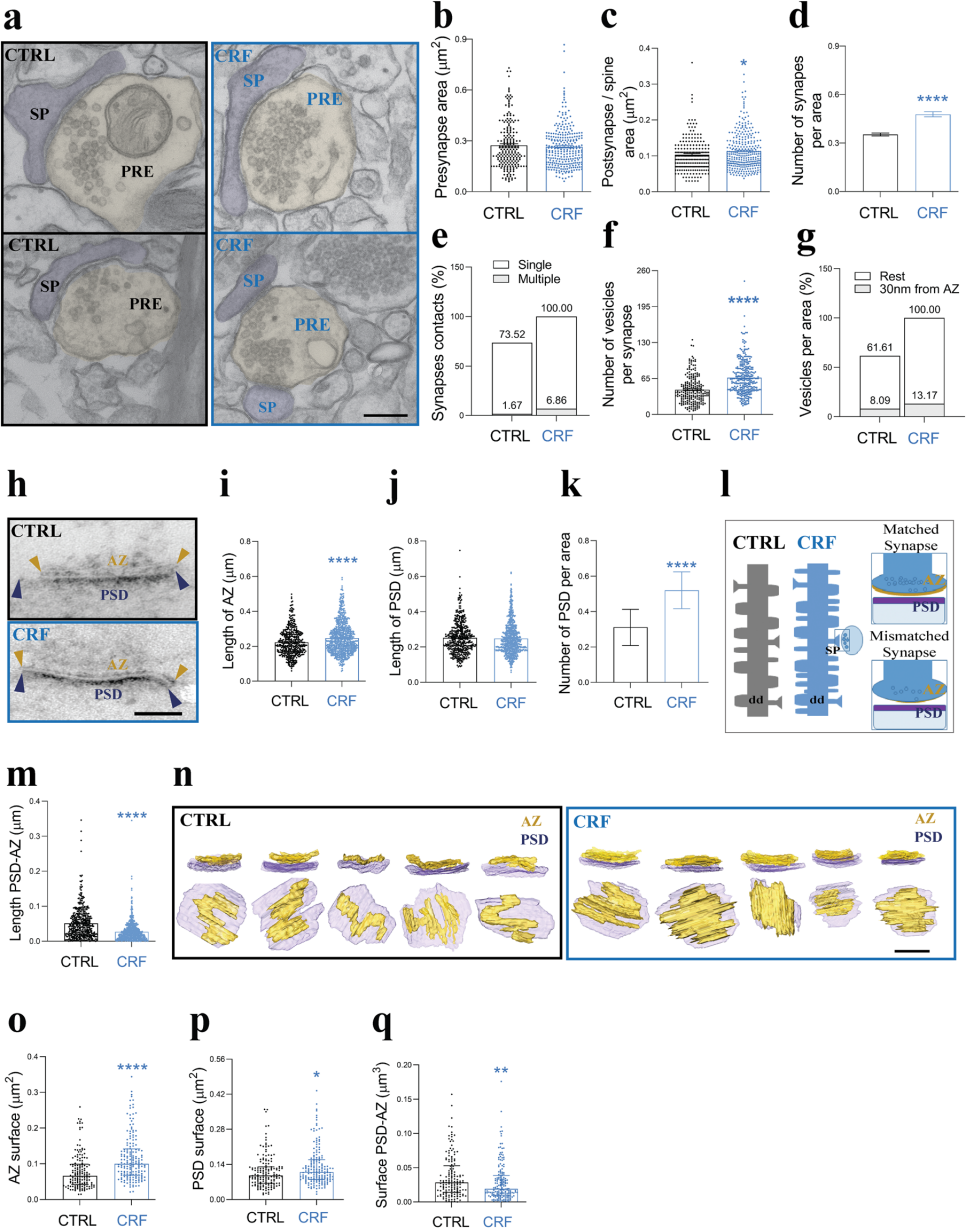
Acute CRF alters multiple aspects of synaptic architecture. **a** TEM images from control (CTRL) and CRF-treated acute slices. Scale bar = 200 nm. **b**, **c** Quantification of presynaptic (PRE), (**b**, shown as median with IQR. CTRL: *N* = 3 animals, *n* = 216 synapses; CRF: *N* = 3, *n* = 283; Mann–Whitney test (U = 30100). *p* = 0.7715), postsynaptic areas (spines, SP) (**c**, shown as median with IQR. CTRL: *N* = 3, *n* = 219 synapses; CRF: *N* = 3, *n* = 304; Mann–Whitney test (U = 29456). *p < 0.05). **d**, **e** CRF (100 nM, 20 min) increased both the number of synapses per area (**d**, shown as median with IQR. CTRL: *N* = 3, *n* = 177 areas; CRF: *N* = 3, *n* = 181; Mann–Whitney test (U = 10819). ****p < 0.0001) and **e** the number of multiple postsynaptic boutons per single presynapse. **f**, **g** CRF increased the total number of vesicles per synapse (**f**, shown as median with IQR. CTRL: *N* = 3, *n* = 220 synapses; CRF: *N* = 3, *n* = 305; Mann–Whitney test (U = 18748). ****p < 0.0001), and **g** reorganized synaptic vesicles towards the active zone (AZ). **h** TEM images of PTA-stained synapses. Scale bar = 100 nm. **i**–**k** CRF increased AZ length (**i**, shown as median with IQR. CTRL: *N* = 3, *n* = 452 synapses; CRF: *N* = 3, *n* = 771; Mann–Whitney test (U = 147985). ****p < 0.0001) and the number of PSD per area (**k**, shown as median with IQR. CTRL: *N* = 3, *n* = 150 areas; CRF: *N* = 3, *n* = 150; Mann–Whitney test (U = 3736). ****p < 0.0001), but not postsynaptic density (PSD) length (**j**, shown as median with IQR. CTRL: *N* = 3, *n* = 452 synapses; CRF: *N* = 3, *n* = 771; Mann–Whitney test (U = 167545). *p* = 0.2610). **l** Relationships between AZ and PSD at the synapse. The upper right represents an idealized, matched synapse, where the lengths of AZ and PSD are approximately equal in length, while the lower a mismatched synapse where the length of the PSD is (typically) larger than the AZ. **m** CRF enhanced synaptic matching between AZ and PSD (shown as median with IQR. CTRL: *N* = 3, *n* = 452 synapses; CRF: *N* = 3, *n* = 771; Mann–Whitney test (U = 109620). ****p < 0.0001). **n** 3D reconstruction of AZ and PSD after FIB-SEM imaging shows a CFR dependent increase in AZ surface area (yellow). Scale bar = 150 nm. **o**–**q** Quantification of AZ-PSD complexity using 3D-reconstructed images, confirms CRF increased AZ surface area (**o**, shown as median with IQR. CTRL: *N* = 3, *n* = 150 synapses; CRF: *N* = 3, *n* = 173; Mann–Whitney test (U = 8026). ****p < 0.0001), increased PSD surface area (**p**, shown as median with IQR. CTRL: *N* = 3, *n* = 150 synapses; CRF: *N* = 3, *n* = 173; Mann–Whitney test (U = 10922). *p < 0.05) and promoted tighter matching between them (**q**, shown as median with IQR. CTRL: *N* = 3, *n* = 150 synapses; CRF: *N* = 3, *n* = 173; Mann–Whitney test (U = 10737). **p < 0.01).

To further study the short-term effects of CRF on synaptic structure and organization, we performed ultrastructural analysis on hippocampal ex vivo acute slices, focusing on synaptic connections on CA1 PCs in the SR, where secondary and tertiary dendrites of PCs are situated and SC synapses are predominantly located (Fig. 5, Supplementary Video 2, 3).

CRF did not affect the presynaptic bouton area (Fig. 5a, b). However, we did observe an increase in postsynaptic compartment size (Fig. 5c), the number of synapses per area unit (Fig. 5d), and number of single presynaptic terminals innervating multiple spines (Fig. 5e). To investigate whether CRF induces structural changes within presynaptic terminals, we analyzed the number and localization of synaptic vesicles. Indeed, CRF increases the total number of vesicles per synapse area and in addition rearranges these vesicles towards the release sites, resulting in more vesicles within 30 nm from the active zone (AZ; Fig. 5f, g). Furthermore, in vivo stress experiments showed an increase in the presynaptic and postsynaptic area, number of synapses, number of single presynaptic terminals innervating multiple spines, and the increase and redistribution of synaptic vesicles (Supplementary Fig. 4).

To evaluate the spatial relationship between AZ and postsynaptic density (PSD), we stained slices with PTA, which highlights macromolecular complexes of AZ/PSD in the synapse [60, 61]. Using our highly modified PTA protocol, we focused on asymmetric synapses at the CA1-SR (Fig. 5h–k). CRF induced a significant increase in the number PSDs and in the length of AZ, without alterations in PSD length. These findings prompted us to investigate the alignment between the AZ and PSD (Fig. 5l). In a “matching” synapse, the size of the AZ and PSD are comparable to each other (Fig. 5l, top), whereas in “mismatched” synapses there is a difference between those two elements (Fig. 5l, bottom). Using this approach, we found CRF significantly increased matching between AZ and PSD length (Fig. 5m). To further examine synapse matching, we utilized FIB-SEM based imaging, to allow a more detailed three-dimensional analysis of synapse ultrastructure (Fig. 5n–q, Supplementary Video 2, 3). The 3D-reconstructed spatial organization of AZ-PSD complexes confirmed a CRF-induced increase in AZ surface, but also showed the previously missed increase in PSD size. In addition, we confirmed that CRF signaling facilitated AZ-PSD matching (Fig. 5n, q).

## Discussion

Acute stress has a diverse range of beneficial effects on brain function [62, 63] and multiple studies have demonstrated the involvement of CRF as a central regulator in this adaptive process [12, 64–67]. However, the acute role of CRF as a local neuromodulator in structural and functional synaptic plasticity has not been investigated extensively. Here, we provide detailed insights into the acute role of CRF as a local neuropeptide in acute stress. Our research shows that the structural and functional consequences of acute stress paradigms can be recapitulated both in vivo and ex vivo, using short-term application of physiologically relevant CRF concentrations [8].

CRF treatment (injected in vivo or applied to acute slices ex vivo) resulted in similar structural adaptations as observed during acute stress paradigms, suggesting a prominent role of CRF in regulating physiological responses to acute stress. Short-term CRF treatment resulted in rapid structural and functional adaptations, leading to an overall increase in functional synaptic contacts. In short, we showed that CRF (1) increased spine density and maturation, (2) increased synapse number and size, (3) revised synaptic vesicle organization towards release sites, (4) enhanced matching of synaptic contact, (5) increased synaptic efficacy and 6) enhanced the functional range of long-term plasticity. Systemically released stress hormones likely cannot be involved in the direct effects that we found after acute stress and CRF application, since these have been reported to reach brain tissue well after the structural and functional changes we describe here [50, 51]. However, there probably is a temporal integration of immediate (initiated by the local release of neuromodulators) and delayed (through systemically derived hormones) stress responses within brain regions. Our in vivo data showed an upregulation of the immediate early gene *crh* and *cfos mRNA* expression (Fig. 1i, j) after acute stress, indicating responses likely also involve widespread long-term changes in neuronal function. Our ex vivo results confirm this local response by treatment of CRF and absence of hormonal response. Together, our findings indicate a prominent role of local released CRF during the immediate phase of acute stress, modulating synaptic input in the CA1 PCs.

Activation of CRF-R1 is required for CRF-induced changes in spine density and maturation, while CRF-R2s are dispensable (Fig. 2b, c). Since CRF-R1 activation is also a prerequisite for inducing *cfos* expression [68, 69], the observed structural changes might depend on processes downstream of *cfos* signaling. Comparably, the transient increase in fEPSP amplitude during CRF applications requires CRF-R1 activation. Since the observed CRF-R1-dependent structural changes would presumably persist after CRF exposure, it seems more likely that the reversible CRF-R1-dependent increase in fEPSP responses is due to a transient increase in presynaptic efficacy via CRF-R1s expressed in the presynaptic compartment [70, 71]. Indeed, both evoked and spontaneous synaptic inputs in CA1 PCs increased while applying CRF, indicating an immediate effect of CRF on synapse function. In contrast, either CRF-R1 or CRF-R2 activation (or both) was sufficient to enhance long-term plasticity (Fig. 4h, i). Our data supports CRF as a positive regulator of synaptic transmission, in agreement with other reports describing CRF generally as a facilitator of excitatory neurotransmission throughout different brain regions [30, 54, 72, 73].

PPF is an activity dependent increase in presynaptic release probability due to accumulation of presynaptic Ca^2+^. CRF increases PPF of the SC synapses (Fig. 3g), likely due to the relocation of synaptic vesicles towards the active zone (Fig. 5g), which would increase either the size or the replenishment rate of the release pool. The CRF-induced increase in the synaptic vesicle number and redistribution towards the active zone is expected to also affect synaptic release efficacy during sustained periods of activity, which is indeed what we observed during train stimulations (Fig. 3i). EM confirmed the increase in the docking pool of vesicles by CRF (Fig. 5f, g), thereby providing evidence of the mechanism of action of CRF in the acute stress response by enhancing structural architecture and functional properties of the synaptic network.

In contrast to CRF-R1, there is still much debate over the presence of CRF-R2 in the rodent hippocampus [7, 10, 74–78]. Some reports confirm expression of CRF-R2 [70, 74, 75, 77], while others claim that the expression is absent or negligible [9, 11, 79]. CRF-R2 mRNA has been reported throughout the hippocampal formation, albeit in lower amounts compared to CRF-R1 [70, 74]. Potentially the presence of different isoforms of CRF-R2 (full-length and truncated) underlies these contradicting reports [9]. In addition, the two receptors are also known to have different kinetics. While CRF-R1 is activated fast in acute stages, studies in knockout mice suggest that CRF signaling via CRF-R2 has a slower kinetic [4, 80, 81]. We found that both CRF receptors were involved in the acute CRF-induced enhancement of synaptic efficacy (as measured by a transient increase in fEPSPs) and played a role in acute CRF signaling.

In conclusion, we report that acute CRF signaling in CA1 PCs involves a complicated interplay of morphological and functional synaptic adaptations, which culminate in enhancing both short- and long-term responsiveness of the underlying neuronal network, potentially affecting hippocampus dependent learning strategies during short stressful events.

## Supplementary information

Supplementary Materials

Supplementary Figure 1

Supplementary Figure 2

Supplementary Figure 3

Supplementary Figure 4

Supplementary Video 1

Supplementary Video 2

Supplementary Video 3

## Data Availability

The datasets generated and analysed during the current study are available from the corresponding author on request.

## References

[1] Selye H. Streß-syndrome. A syndrome produced by diverse nocuos agents. Nature. 1936;138:32. 10.1176/jnp.10.2.230a 9722327

[2] Selye H (1950). Stress and the general adaptation syndrome. Br Med J.

[3] Selye H (1975). Confusion and controversy in the stress field. J Hum Stress.

[4] Joëls M, Baram TZ (2009). The neuro-symphony of stress. Nat Rev Neurosci.

[5] Mcewen BS, Gianaros PJ (2010). Central role of the brain in stress and adaptation: links to socioeconomic status, health, and disease. Ann N Y Acad Sci.

[6] Chrousos GP (2009). Stress and disorders of the stress system. Nat Rev Endocrinol.

[7] Maras PM, Baram TZ (2012). Sculpting the hippocampus from within: stress, spines, and CRH. Trends Neurosci.

[8] Deussing JM, Chen AA (2018). The corticotropin-releasing factor family: physiology of the stress response. Physiol Rev.

[9] Henckens MJ, Deussing JM, Chen A (2016). Region-specific roles of the corticotropin-releasing factor-urocortin system in stress. Nat Rev Neurosci.

[10] Dedic N, Deussing AC, Jan M (2017). The CRF family of neuropeptides and their receptors—-mediators of the central stress response. Curr Mol Pharmacol.

[11] Vandael D, Gounko NV (2019). Corticotropin releasing factor-binding protein (CRF-BP) as a potential new therapeutic target in Alzheimer’s disease and stress disorders. Transl Psychiatry.

[12] Chen Y, Andres AL, Frotscher M, Baram TZ (2012). Tuning synaptic transmission in the hippocampus by stress: the CRH system. Front Cell Neurosci.

[13] Gunn BG, Sanchez GA, Lynch G, Baram TZ, Chen Y (2019). Hyper-diversity of CRH interneurons in mouse hippocampus. Brain Struct. Funct.

[14] Chen Y, Brunson KL, Müller MB, Cariaga W, Baram TZ (2000). Immunocytochemical distribution of corticotropin-releasing hormone receptor type-1 (CRF1)-like immunoreactivity in the mouse brain: light microscopy analysis using an antibody directed against the C-terminus. J Comp Neurol.

[15] Refojo D, Echenique C, Müller MB, Reul JM, Deussing JM, Wurst W (2005). Corticotropin-releasing hormone activates ERK1/2 MAPK in specific brain areas. Proc Natl Acad Sci USA.

[16] McEwen BS, Gianaros PJ (2010). Stress- and allostasis-induced brain plasticity. Annu Rev Med.

[17] Zoladz PR, Diamond DM (2009). Linear and non-linear dose-response functions reveal a hormetic relationship between stress and learning. Dose Response.

[18] Schwabe L, Joëls M, Roozendaal B, Wolf OT, Oitzl MS (2012). Stress effects on memory: an update and integration. Neurosci Biobehav Rev.

[19] Krugers HJ, Lucassen PJ, Karst H, Joëls M (2010). Chronic stress effects on hippocampal structure and synaptic function: Relevance for depression and normalization by anti-glucocorticoid treatment. Front Synaptic Neurosci.

[20] De Quervain DJF, Roozendaal B, McGaugh JL (1998). Stress and glucocorticoids impair retrieval of long-term spatial memory. Nature..

[21] Justice NJ (2018). The relationship between stress and Alzheimer’s disease. Neurobiol Stress.

[22] Gounko NV, Swinny JD, Kalicharan D, Jafari S, Corteen N, Seifi M (2013). Corticotropin-releasing factor and urocortin regulate spine and synapse formation: structural basis for stress-induced neuronal remodeling and pathology. Mol Psychiatry.

[23] Chen Y, Kramár EA, Chen LY, Babayan AH, Andres AL, Gall CM (2013). Impairment of synaptic plasticity by the stress mediator CRH involves selective destruction of thin dendritic spines via RhoA signaling. Mol Psychiatry.

[24] Joëls M, Fernandez G, Roozendaal B (2011). Stress and emotional memory: a matter of timing. Trends Cogn Sci.

[25] Swinny JD, Metzger F, IJkema-Paassen J, Gounko NV, Gramsbergen A, van der Want JJ (2004). Corticotropin-releasing factor and urocortin differentially modulate rat Purkinje cell dendritic outgrowth and differentiation in vitro. Eur J Neurosci.

[26] Blank T, Nijholt I, Eckart K, Spiess J (2002). Priming of long-term potentiation in mouse hippocampus by corticotropin-releasing factor and acute stress: implications for hippocampus-dependent learning. J Neurosci.

[27] Rebaudo R, Melani R, Balestrino M, Izvarina N (2001). Electrophysiological effects of sustained delivery of CRF and its receptor agonists in hippocampal slices. Brain Res.

[28] Chen Y, Rex CS, Rice CJ, Dubé CM, Gall CM, Lynch G (2010). Correlated memory defects and hippocampal dendritic spine loss after acute stress involve corticotropin-releasing hormone signaling. Proc Natl Acad Sci USA.

[29] Chen Y, Bender RA, Brunson KL, Pomper JK, Grigoriadis DE, Wurst W (2004). Modulation of dendritic differentiation by corticotropin-releasing factor in the developing hippocampus. Proc Natl Acad Sci USA.

[30] Aldenhoff JB, Gruol DL, Rivier J, Vale W, Siggins GR (1983). Corticotropin releasing factor decreases postburst hyperpolarizations and excites hippocampal neurons. Science.

[31] Haug T, Storm JF (2000). Protein kinase A mediates the modulation of the slow Ca2+-dependent K+ current, I(sAHP), by the neuropeptides CRF, VIP, and CGRP in hippocampal pyramidal neurons. J. Neurophysiol.

[32] Bali A, Jaggi AS (2015). Electric foot shock stress: a useful tool in neuropsychiatric studies. Rev Neurosci.

[33] Clark SM, Sand J, Francis TC, Nagaraju A, Michael KC, Keegan AD (2014). Immune status influences fear and anxiety responses in mice after acute stress exposure. Brain Behav Immun.

[34] Wu YP, Gao HY, Ouyang SH, Kurihara H, He RR, Li YF (2019). Predator stress-induced depression is associated with inhibition of hippocampal neurogenesis in adult male mice. Neural Regen Res.

[35] Sterley TL, Baimoukhametova D, Füzesi T, Zurek AA, Daviu N, Rasiah NP (2018). Social transmission and buffering of synaptic changes after stress. Nat Neurosci.

[36] Wilcock DM, DiCarlo G, Henderson D, Jackson J, Clarke K, Ugen KE (2003). Intracranially administered anti-Aβ antibodies reduce β-amyloid deposition by mechanisms both independent of and associated with microglial activation. J Neurosci.

[37] Belevich I, Joensuu M, Kumar D, Vihinen H, Jokitalo E (2016). Microscopy image browser: a platform for segmentation and analysis of multidimensional datasets. PLoS Biol.

[38] Condomitti G, Wierda KD, Schroeder A, Rubio SE, Vennekens KM, Orlandi C (2018). An input-specific orphan receptor GPR158-HSPG interaction organizes hippocampal mossy fiber-CA3 synapses. Neuron..

[39] Pastoll H, White M, Nolan M. Preparation of parasagittal slices for the investigation of dorsal-ventral organization of the rodent medial entorhinal cortex. J Vis Exp. 2012;6:17. 10.3791/3802 PMC3460585 22491152

[40] Largo-Barrientos P, Apóstolo N, Creemers E, Callaerts-Vegh Z, Swerts J, Davies C (2021). Lowering Synaptogyrin-3 expression rescues Tau-induced memory defects and synaptic loss in the presence of microglial activation. Neuron.

[41] Bessa JM, Ferreira D, Melo I, Marques F, Cerqueira JJ, Palha JA (2009). The mood-improving actions of antidepressants do not depend on neurogenesis but are associated with neuronal remodeling. Mol Psychiatry.

[42] Leuner B, Shors TJ (2013). Stress, anxiety, and dendritic spines: what are the connections?. Neuroscience.

[43] Sandi C, Davies HA, Cordero MI, Rodriguez JJ, Popov VI, Stewart MG (2003). Rapid reversal of stress induced loss of synapses in CA3 of rat hippocampus following water maze training. Eur J Neurosci.

[44] Magariños AM, García Verdugo JM, Mcewen BS (1997). Chronic stress alters synaptic terminal structure in hippocampus. Proc Natl Acad Sci USA.

[45] Gong S, Miao YL, Jiao GZ, Sun MJ, Li H, Lin J (2015). Dynamics and correlation of serum cortisol and corticosterone under different physiological or stressful conditions in mice. PLoS ONE.

[46] Mcgill BE, Bundle SF, Yaylaoglu MB, Carson JP, Thaller C, Zoghbi HY (2006). Enhanced anxiety and stress-induced corticosterone release are associated with increased Crh expression in a mouse model of Rett syndrome. Proc Natl Acad Sci USA.

[47] Mcclennen SJ, Cortright DN, Seasholtz AF (1998). Regulation of pituitary corticotropin-releasing hormone-binding protein messenger ribonucleic acid levels by restraint stress and adrenalectomy. Endocrinology.

[48] Hering H, Sheng M (2001). Dentritic spines: structure, dynamics and regulation. Nat Rev Neurosci.

[49] Segal M (2005). Dendritic spines and long-term plasticity. Nat Rev Neurosci.

[50] Droste SK, de Groote L, Atkinson HC, Lightman SL, Reul JM, Linthorst AC (2008). Corticosterone levels in the brain show a distinct ultradian rhythm but a delayed response to forced swim stress. Endocrinology.

[51] Qian X, Droste SK, Gutièrrez-Mecinas M, Collins A, Kersanté F, Reul JM (2011). A rapid release of corticosteroid-binding globulin from the liver restrains the glucocorticoid hormone response to acute stress. Endocrinology.

[52] Salvatore M, Wiersielis KR, Luz S, Waxler DE, Bhatnagar S, Bangasser DA (2018). Sex differences in circuits activated by corticotropin releasing factor in rats. Horm Behav.

[53] Boutillier AL, Sassone-Corsi P, Loeffler JP (1991). The Protooncogene c-fos is induced by corticotropin-releasing factor and stimulates proopiomelanocortin gene transcription in pituitary cells. Mol Endocrinol.

[54] Dedic N, Kühne C, Gomes KS, Hartmann J, Ressler KJ, Schmidt MV (2019). Deletion of CRH from GABAergic forebrain neurons promotes stress resilience and dampens stress-induced changes in neuronal activity. Front Neurosci.

[55] Henckens MJAG, Printz Y, Shamgar U, Dine J, Lebow M, Drori Y (2017). CRF receptor type 2 neurons in the posterior bed nucleus of the stria terminalis critically contribute to stress recovery. Mol Psychiatry.

[56] Miyata M, Okada D, Hashimoto K, Kano M, Ito M (1999). Corticotropin-releasing factor plays a permissive role in cerebellar long-term depression. Neuron.

[57] Wong TP, Howland JG, Robillard JM, Ge Y, Yu W, Titterness AK (2007). Hippocampal long-term depression mediates acute stress-induced spatial memory retrieval impairment. Proc Natl Acad Sci USA.

[58] Sumi T, Harada K (2020). Mechanism underlying hippocampal long-term potentiation and depression based on competition between endocytosis and exocytosis of AMPA receptors. Sci Rep.

[59] Lemon N, Manahan-Vaughan D (2012). Dopamine D1/D5 receptors contribute to de novo hippocampal LTD mediated by novel spatial exploration or locus coeruleus activity. Cereb Cortex.

[60] Gray EG (1966). Problems of Interpreting the Fine Structure of Vertebrate and Invertebrate Synapses. International Review General & Experimental Zoology.

[61] Bloom FE, Aghajanian GK (1968). Fine structural and cytochemical analysis of the staining of synaptic junctions with phosphotungstic acid. J Ultrasructure Res.

[62] Yuen EY, Liu W, Karatsoreos IN, Feng J, McEwen BS, Yan Z (2009). Acute stress enhances glutamatergic transmission in prefrontal cortex and facilitates working memory. Proc Natl Acad Sci USA.

[63] Parihar VK, Hattiangady B, Kuruba R, Shuai B, Shetty AK (2011). Predictable chronic mild stress improves mood, hippocampal neurogenesis and memory. Mol Psychiatry.

[64] Adzic M, Djordjevic J, Djordjevic A, Niciforovic A, Demonacos C, Radojcic M (2009). Acute or chronic stress induce cell compartment-specific phosphorylation of glucocorticoid receptor and alter its transcriptional activity in Wistar rat brain. J Endocrinol.

[65] Chen Y, Brunson KL, Adelmann G, Bender RA, Frotscher M, Baram TZ (2004). Hippocampal corticotropin releasing hormone: pre- and postsynaptic location and release by stress. Neuroscience..

[66] Aguilar-Valles A, Sánchez E, de Gortari P, Balderas I, Ramírez-Amaya V, Bermúdez-Rattoni F (2006). Analysis of the stress response in rats trained in the water-maze: differential expression of corticotropin-releasing hormone, CRH-R1, glucocorticoid receptors and brain-derived neurotrophic factor in limbic regions. Neuroendocrinology.

[67] Jiang Z, Rajamanickam S, Justice NJ (2019). CRF signaling between neurons in the paraventricular nucleus of the hypothalamus (PVN) coordinates stress responses. Neurobiol Stress.

[68] Doyon C, Samson P, Lalonde J, Richard D (2007). Effects of the CRF1 receptor antagonist SSR125543 on energy balance and food deprivation-induced neuronal activation in obese Zucker rats. J Endocrinol.

[69] Skórzewska A, Bidziński A, Hamed A, Lehner M, Turzyńska D, Sobolewska A (2008). The influence of CRF and α-helical CRF(9-41) on rat fear responses, c-Fos and CRF expression, and concentration of amino acids in brain structures. Horm Behav.

[70] Gunn BG, Cox CD, Chen Y, Frotscher M, Gall CM, Baram TZ (2017). The endogenous stress hormone CRH modulates excitatory transmission and network physiology in hippocampus. Cereb Cortex.

[71] Bajo M, Cruz MT, Siggins GR, Messing R, Roberto M (2008). Protein kinase C epsilon mediation of CRF- and ethanol-induced GABA release in central amygdala. Proc Natl Acad Sci USA.

[72] Hollrigel GS, Chen K, Baram TZ, Soltesz I (1998). The pro-convulsant actions of corticotropin-releasing hormone in the hippocampus of infant rats. Neuroscience..

[73] Giesbrecht CJ, Mackay JP, Silveira HB, Urban JH, Colmers WF (2010). Countervailing modulation of Ihby neuropeptide Y and corticotrophin-releasing factor in basolateral amygdala as a possible mechanism for their effects on stress-related behaviors. J Neurosci.

[74] Van Pett K, Viau V, Bittencourt JC, Chan RK, Li HY, Arias C (2000). Distribution of mRNAs encoding CRF receptors in brain and pituitary of rat and mouse. J Comp Neurol..

[75] Hiroi N, Wong ML, Licinio J, Park C, Young M, Gold PW (2001). Expression of corticotropin releasing hormone receptors type I and type II mRNA in suicide victims and controls. Mol Psychiatry.

[76] Gunn BG, Baram TZ (2017). Stress and seizures: space, time and hippocampal circuits. Trends Neurosci.

[77] Carboni L, Romoli B, Bate ST, Romualdi P, Zoli M (2018). Increased expression of CRF and CRF-receptors in dorsal striatum, hippocampus, and prefrontal cortex after the development of nicotine sensitization in rats. Drug Alcohol Depend.

[78] Smith GW, Aubry JM, Dellu F, Contarino A, Bilezikjian LM, Gold LH (1998). Corticotropin releasing factor receptor 1–deficient mice display decreased anxiety, impaired stress response, and aberrant neuroendocrine development. Neuron.

[79] Bagosi Z, Balangó B, Pintér D, Csabafi K, Jászberényi M, Szabó G (2015). The effects of CRF and urocortins on the hippocampal glutamate release. Neurochem Int.

[80] Bale TL, Contarino A, Smith GW, Chan R, Gold LH, Sawchenko PE (2000). Mice deficient for corticotropin-releasing hormone receptor-2 display anxiety-like behaviour and are hypersensitive to stress. Nat Genet.

[81] Coste SC, Kesterson RA, Heldwein KA, Stevens SL, Heard AD, Hollis JH (2000). Abnormal adaptations to stress and impaired cardiovascular function in mice lacking corticotropin-releasing hormone receptor-2. Nat Genet.

