# Supplementary material for: Corticotropin-releasing factor induces functional and structural synaptic remodelling in acute stress
Source: Transl Psychiatry. 2021 Jul 7;11:378. doi: 10.1038/s41398-021-01497-2 (PMC8263770; doi:10.1038/s41398-021-01497-2)
Supplement: Supplementary file 1 — Supplementary Materials [file 41398_2021_1497_MOESM1_ESM.docx]

Supplementary Materials

**Basescope Probes**

| **Gene** | **Genbank Accession** | **Sequence** | **Probe design number** |
| --- | --- | --- | --- |
| Mouse c-fos oncogene | NM_010234 | aagtc tgcgttgcag actgagattg ccaatctgct gaaagagaag gaaaaactgg agtttatttt ggcagcccac cgacctgcct gcaagatccc cgatgacctt ggcttcccag aggagatgtc t | NPR-0008898 |
| Mus musculus preprocorticotropin-releasing hormone gene | AY128673.1 | gcagttagct cagcaagctc acagcaacag gaaactgatg gagattatcg ggaaatgaaa tgttgcgctt ggccaaaacg attctgcatt tagcacacaa gtaaaaataa aaatttaaaa cacagtattc tgtaccatat cgc | NPR-0022460 |

**Supplementary Figure 1 Legend. Blood plasma levels in acute stress paradigms. a-b** Acute stress exhibited no difference of corticosterone concentrations in plasma compared to control animals (basal). Quantification of corticosterone concentrations in blood plasma after foot shock (FS) (**a,** shown as mean±SEM, CTRL: N=9 animals; FS: N=12; unpaired t-test (t=1535, df=19). p=0.1413) and predator odor (PO) (**b**, shown as median with IQR, CTRL: N=4; PO: N=4; Mann-Whitney test (U=2). p=0.1143).

**Supplementary Figure 2 Legend. Acute CRF leads to hours-long lasting spine growth in hippocampal PC CA1 *ex vivo* (Thy1YFP mice).** Quantifications of spines from CA1 proximal dendrites in CTRL condition, after 30 minutes of CRF 100nM for 20 minutes and after 30, 60, 120 minutes wash out (shown as median with IQR. CTRL: N=4 animals, n=13 cells, d=21 dendrites; CRF: N=4, n=11, d=21; wash out 30 minutes: N=4, n=10, d=17; wash out 60 minutes: N=4, n=11, d=21 and wash out 120 minutes: N=4, n=12, d=27; Kruskal-Wallis analysis of variance followed by Dunn’s multiple comparison test (Kruskal-Wallis statistic=24.60). **P<0.05, ***P<0.0001).

**Supplementary Figure 3 Legend. Acute CRF exposure increases calcium release *in vivo* in CA1 PCs in slices prepared from mice expressing the green fluorescent calcium indicator, GCaMP6 (Thy1-GCaMP6 mice). a** Image of PCs CA1 before treatment with CRF (left) and same field of view after 15 minutes with CRF 100 nM (right). Arrows indicate same cells in control and CRF conditions. **b** The traces of calcium influx of individual cells marked with arrows as in a, before (black) and after CRF application (blue). N=4 animals. Scale bar 50µm.

**Supplementary Figure 4 Legend. Stress alters the synaptic architecture. a** TEM images from control (CTRL) and stress animals (PRE - presynaptic, SP – spine/postsynaptic). Scale bar=200nm. **b-c** Quantification of presynaptic (shown as median with IQR. CTRL: N=2 animals, n=145 synapses; Stress: N=2, n=169; Mann-Whitney test (U=10212). **p<0.01) (b) and postsynaptic (c) compartments areas (shown as median with IQR. CTRL: N=2, n=145 synapses; Stress: N=2, n=169; Mann-Whitney test (U=9112). ***p<0.0001). **d-e** The number of synapses per area increased due to stress (**d**, CTRL: N=2,

n=121 areas; Stress: N=2, n=118; Mann-Whitney test (U=6118). *p<0.05), as well as the number of multiple postsynaptic boutons (in grey) per single presynapse, **e**. **f-g** Number of vesicles per synapse (**f**, shown as median with IQR. CTRL: N=2, n=145 synapses; Stress: N=2, n=171; Mann-Whitney test (U=9338). ****p<0.0001), **g** the number of vesicles within 30nm of the active zone (AZ) “docked” (in gray) and remaining vesicles (white) per synaptic area increased due to stress.

**Supplementary Video 1 Legend. Acute CRF exposure increases calcium release in CA1 PC layer *in vivo,* in slices prepared from mice expressing the green fluorescent calcium indicator, GCaMP6 (Thy1-GCaMP6 mice).** Left, two-photon microscope imaging using 20x objective started in aCSF, with a capture of 300 images (average of 15 frames per image), with a 30 ms interval. After 300 images, slice was continue perfused with 100 nM CRF in aCSF, and other 300 images were taken. Right, after 15 minutes with CRF in aCSF, the same field of view was imaged last time, 600 images were taken with the same settings. Arrows indicate same cells in control and CRF condition. Abbreviations: aCSF-artificial cerebrospinal fluid, Ca- calcium, CTRL - control, CRF - corticotropin-releasing factor.

**Supplementary Video 2 Legend. FIB-SEM imaging and reconstruction of PTA staining in control hippocampal slices**. The FIB-SEM was set to remove 5-nm-thick layers and image acquisition was done using a backscattered electron detector at 1.5 kV (0.005 µm/pixel), at 5kX magnification. Individual segmentation of an AZ shown in yellow and PSD segment in purple (N=1). Abbreviations: AZ - active zone, CTRL - control, CRF - corticotropin-releasing factor, PSD - postsynaptic density, PTA - phosphotungstic acid.

**Supplementary Video 3 Legend. FIB-SEM imaging and reconstruction of PTA staining in CRF treated hippocampal slices.** The FIB-SEM was set to remove 5-nm-thick layers and image acquisition was done using a backscattered electron detector at 1.5 kV (0.005 µm/pixel), at 5kX magnification. Individual segmentation of an AZ shown in yellow and PSD segment in purple (N=1). Abbreviations: AZ - active zone, CTRL - control, CRF - corticotropin-releasing factor, PSD - postsynaptic density, PTA - phosphotungstic acid.
