# Supplementary figures and images for: Corticotropin-releasing factor induces functional and structural synaptic remodelling in acute stress
Source: Transl Psychiatry. 2021 Jul 7;11:378. doi: 10.1038/s41398-021-01497-2 (PMC8263770; doi:10.1038/s41398-021-01497-2)

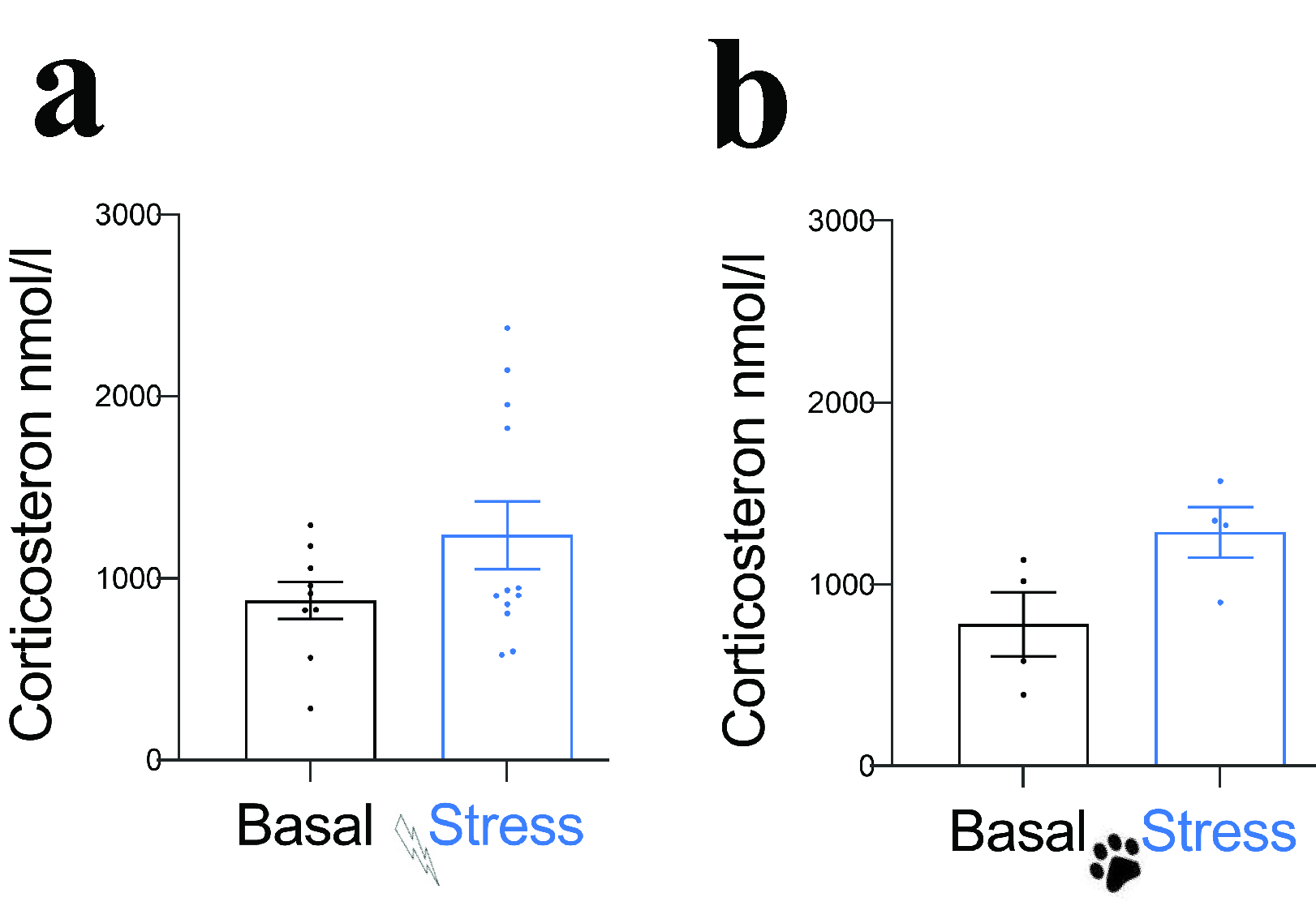

Supplement: Supplementary file 2 — Supplementary Figure 1 [file 41398_2021_1497_MOESM2_ESM.tif]

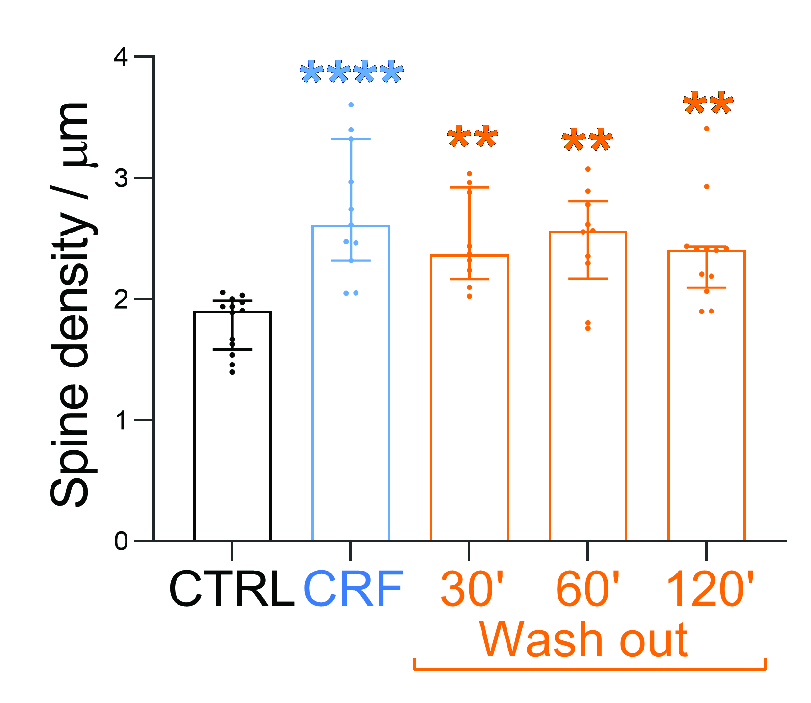

Supplement: Supplementary file 3 — Supplementary Figure 2 [file 41398_2021_1497_MOESM3_ESM.tif]

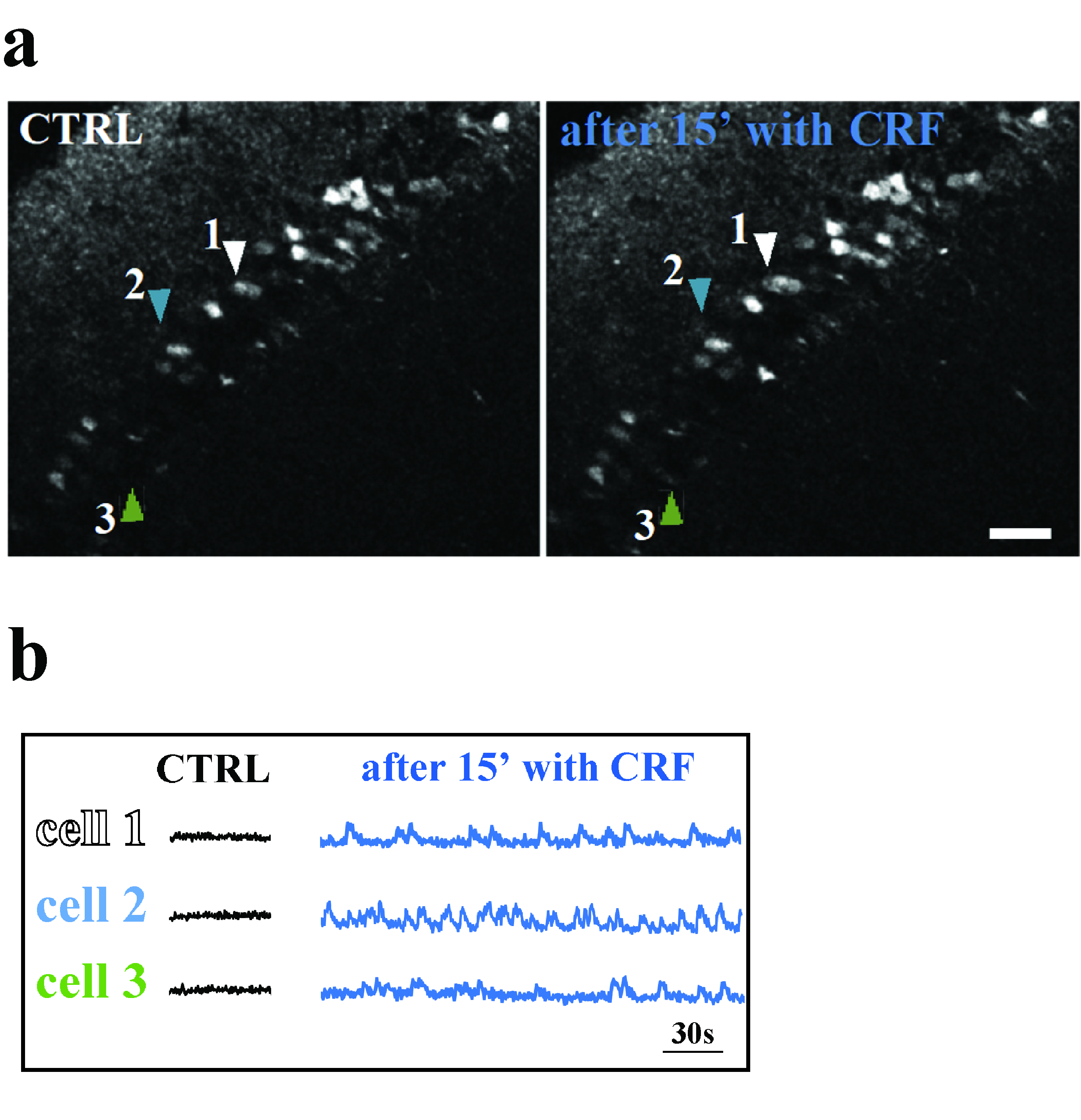

Supplement: Supplementary file 4 — Supplementary Figure 3 [file 41398_2021_1497_MOESM4_ESM.tif]

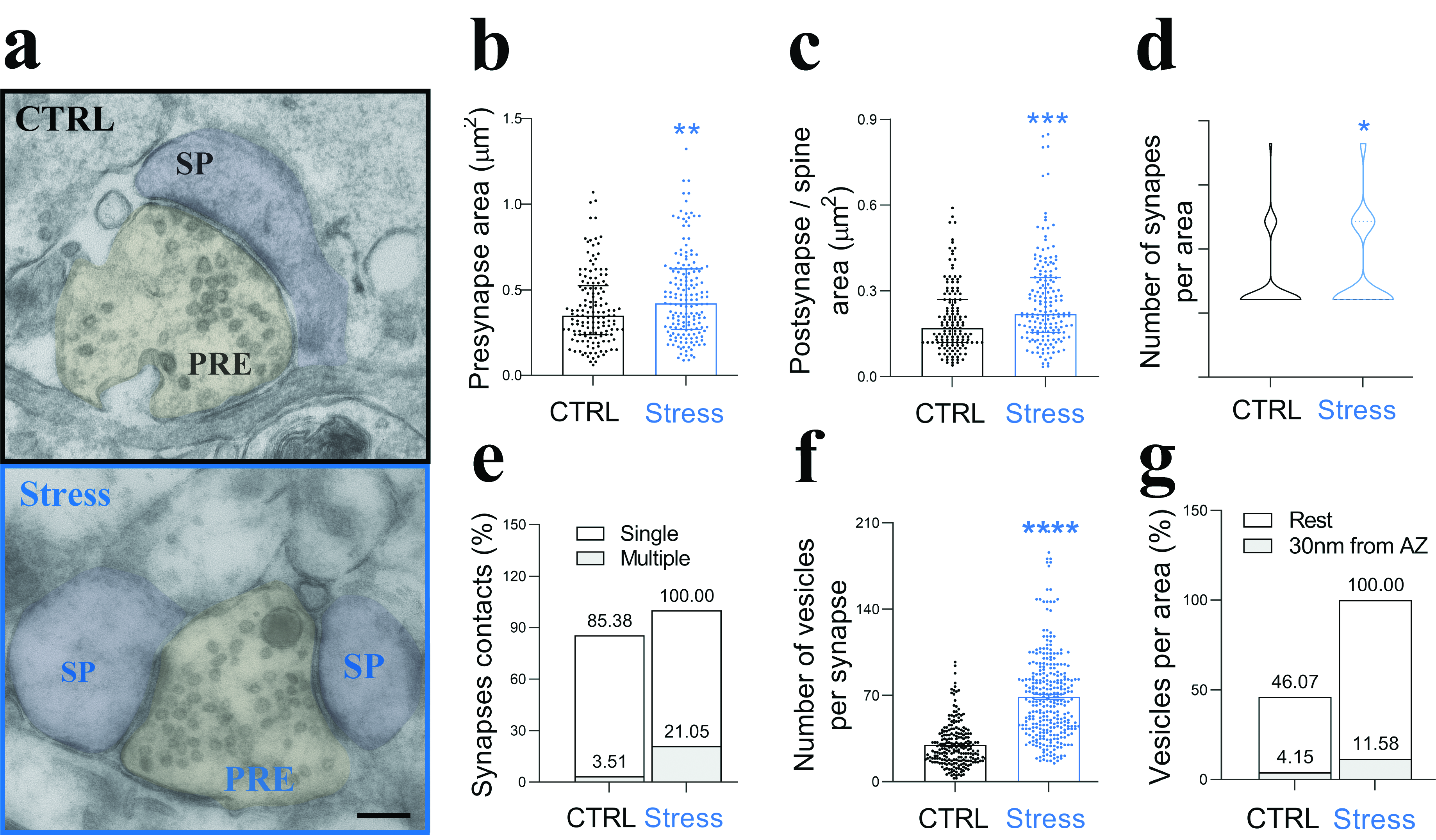

Supplement: Supplementary file 5 — Supplementary Figure 4 [file 41398_2021_1497_MOESM5_ESM.tif]
